# Characterization of a Potential Therapeutic Anti-Canine PD-1 Single Domain Antibody Produced in Yeast

**DOI:** 10.3390/vetsci12070649

**Published:** 2025-07-08

**Authors:** Kartikeya Vijayasimha, Andrew J. Annalora, Dan V. Mourich, Carl E. Ruby, Brian P. Dolan, Laura Crowell, Vu Ha Minh Le, Maureen K. Larson, Shay Bracha, Christopher K. Cebra

**Affiliations:** 1 Department of Clinical Sciences, Oregon State University, Corvallis, OR 97331, USA; vijayask@oregonstate.edu (K.V.); brian.dolan@oregonstate.edu (B.P.D.); 2 Department of Biomedical Sciences, Oregon State University, Corvallis, OR 97331, USA; 3 Department of Environmental and Molecular Toxicology, Oregon State University, Corvallis, OR 97331, USA; andrew.annalora@oregonstate.edu; 4 Department of Clinical Science, Carlson College of Veterinary Medicine, Oregon State University, Corvallis, OR 97331, USA; carl.ruby@oregonstate.edu (C.E.R.); maureen.larson@oregonstate.edu (M.K.L.); 5 Sunflower Therapeutics, Medford, MA 02155, USA; laura@sunflowertx.com (L.C.); levuhaminh96@gmail.com (V.H.M.L.); 6 Department of Veterinary Clinical Sciences, College of Veterinary Medicine, The Ohio State University, Columbus, OH 97331, USA; bracha.2@osu.edu

**Keywords:** immunotherapeutic, PD-1, single domain antibody, yeast, canine, cancer

## Abstract

A single domain antibody (SDAb) targeting canine PD-1 was developed as a potential immunotherapeutic for canine cancer. An alpaca was immunized with canine PD-1 protein, and a phage-display library was constructed using mRNA isolated from peripheral lymphocytes. Screening of the library yielded multiple SDAb candidates capable of nanomolar binding to canine PD-1. Among these, clone STX-1b5 demonstrated high expression in a yeast-based recombinant system and was selected for further characterization. Binding and competition assays using ELISA confirmed its ability to bind canine PD-1 and block PDL-1 interaction. In silico structural modeling supported the interaction of STX-1b5 with key PD-1 residues implicated in ligand binding. These findings support the feasibility of using SDAbs and cost-effective yeast expression systems to generate immunotherapeutics for veterinary use, with STX-1b5 representing a promising lead candidate for future clinical development.

## Simple Summary

Cancer is the leading cause of death in dogs, with one in three dogs expected to die of cancer. Although promising in the treatment of many human cancers, monoclonal antibodies (MAb) as immunotherapeutics are costly and exhibit poor transferability between species, inhibiting their use in veterinary clinics. Nanobodies are single-domain antibodies (SDAb) that share the same antigen specificity as Mab but have added benefits, including high modularity, allowing use in a variety of protein expression systems that facilitate desirable recombinant modifications to the primary protein sequence. We describe the generation, discovery, production, and functional characterization of an SDAb against canine PD-1 (caPD1) using alpaca immunization and phage display library antigen selection. We further show the amenability of our SDAb to production by a yeast expression system, illustrating a potential path for the advancement of functional, low-cost immunotherapeutics in veterinary medicine.

## 1. Introduction

Cancer is the leading cause of death in dogs in the United States [1], with a one in three probability of a dog succumbing to cancer [2,3]. Despite this, current canine cancer treatments continue to follow decades-old human treatment protocols that include cytotoxic chemotherapy, radiotherapy, and surgery. These methods frequently fail to effectively control the disease and are associated with significant side effects [4]. There is an urgent need for more modern, effective, and safer approaches.

In human medicine, advances in immunotherapy have revolutionized cancer care [5]. Immune checkpoint inhibitors (ICI), CAR-T cells, cytokines, and vaccines represent some of the distinct immunotherapeutic modalities. Checkpoint inhibitors were among the first to show improved overall survival [6,7,8], with those targeting inhibitory pathways involved in anti-cancer CD8+ T cell quiescence and death appearing to be particularly efficacious [9]. One therapeutic example is antibody-based blockade of programmed cell death protein 1 (PD-1) interaction with its ligand, PD-L1. PD-1 is an inhibitory receptor expressed on activated T cells; binding PD-L1 inhibits further activation and promotes apoptosis of the T-cell [10]. Macrophages, activated lymphocytes, dendritic cells, and some epithelial cells are normal expressers of PD-L1 [11], and it likely plays a role in preventing autoimmune disease. However, several types of tumor cells also express PD-L1 [12,13,14], thereby limiting the T-cell response against tumor antigens [10]. Monoclonal antibodies have been used as a treatment, blocking PD1/PDL1 binding to prevent this immune evasion [15]. This has been shown to increase the remission for metastatic cancers by up to 20%, and one such agent is currently the top-selling pharmaceutical agent for people in the world.

Dogs could greatly benefit from access to modern and effective cancer immunotherapies. However, most monoclonal antibodies developed for human use are not suitable for dogs due to both high costs and species-specific Fc antigens. Targeting the PD-1/PD-L1 checkpoint, which functions similarly in dogs as it does in humans [16,17,18,19], is particularly promising. Several monoclonal antibodies targeting canine PD-1 are currently in development [20,21,22]. Despite this, monoclonal antibodies have several drawbacks, such as a high rate of complications, unpredictable clinical responses, and significant development and production costs [23]. Therefore, utilizing additional therapeutic platforms to address these limitations may enhance canine cancer therapy.

Single-domain antibody (SDAb) fragments, also known as nanobodies, derived from camelid heavy chain antibodies, may offer an advantageous alternative drug development approach [13,24,25]. Consisting of a single independent variable domain (VHH), these fragments possess similar target specificity to monoclonal antibodies [26]. However, they are smaller, more stable, have better penetration in the tumor microenvironment, and exhibit low immunogenicity [27,28]. The absence of the Fc region significantly reduces the risk of unintended immune stimulation and allows for cross-species application. Additionally, their smaller size and simpler structure facilitate efficient production in microbial expression systems, which can provide notable advantages over mammalian cell line-based expression [29]. For instance, yeast-based systems are simpler, faster, and more scalable for commercial production [30]. Yeast also offers benefits over prokaryotic expression, including proper protein folding and post-translational modification. The SDAb platform is relatively new and has not been extensively explored in the veterinary field [31,32].

Our objective is to demonstrate an alternative pathway for developing innovative cancer immunotherapeutics for dogs. Here we describe the isolation of an anti-PD1 SDAb using a phage display library, followed by the optimization of candidates for expression and production in a commercially available yeast expression system. Utilizing a strategy of camelid immunization with canine PD-1 (cPD-1) followed by construction and antigen selection of a phage display library, we isolated several individual anti-canine PD-1 reactive phage clones and determined a candidate sequence that exhibits significant binding to the cPD-1 protein. Validation of blocking functionality of the SDAb was accomplished using competition ELISA to determine the lowest concentration able to maximally outcompete canine PD-L1 binding to canine PD-1 as compared to a validated positive control.

## 2. Materials and Methods

Production of Canine PD-1 for Immunizations. Transfection of COS7 cells was performed by lipofection using Lipofectamine 3000 (Thermo Fisher Scientific Inc., Waltham, MA, USA) according to manufacturer’s instructions. Briefly, 5 × 10^4^ COS-7 cells were grown in one well of a 24-well plate. A 500 mg amount of PCMV3 encoding canine PD-1 (Gene ID: 486213) was diluted in 100 mL opti-MEM and mixed with Lipofectamine reagent. After incubation for 25 min, the mixture was added to wells with COS-7 cells. Cells were allowed to recover for 24 h at 37 °C in a CO_2_ incubator before the addition of 400 μg/mL G418 for the selection of transfected cells. Protein was extracted from cell lysates via detergent-based sonication and subsequent dialysis in phosphate-buffered saline [33].

Alpaca Immunization. A healthy Oregon State University-owned alpaca was the host for SD-antibody immunization. All animal protocols were approved by the Oregon State University Institutional Animal Care and Use Committee (IACUC). To obtain SDAb able to bind to canine PD-1, a healthy adult alpaca was administered 4 rounds (subcutaneous 4 weeks apart) of DNA (2 separate sites X 250 μg/injection) as plasmid pCMV3.1 expression vector encoding canine PD-1 (ACCESSION# AB850882). All plasmid material was purified from bacteria using Endofree plasmid extraction kits (Qiagen. Germantown, MD, USA). DNA was resuspended in PBS buffer (Invitrogen. Carlsbad, CA, USA) at a concentration of 1 mg/mL and stored at −20 °C. Four weeks after the last cDNA injection, 200 μg of purified canine PD-1 His-tagged protein in a total of 500 μL of Sigma Adjuvant Systems (s6322) was administered by subcutaneous injection in the neck region. Purified protein for the vaccine was derived from cultured COS cells transfected with the canine PD-1 construct. Pre-immune serum was collected before the initial cDNA injection and following each immunization and screened for the presence of anti-PD-1 VHH.

Western Blot Analysis for cPD-1 Antigen-Specific Serum Reactivity. Blood was collected at ~24 weeks following initial immunization, and serum was screened for the presence of anti-PD-1 SDAb. Protein lysates from canine PD-1 expressing COS cells and control COS cells were separated on 15% SDS-PAGE gels, transferred to a nitrocellulose membrane, blocked with 5% (*w*/*v*) nonfat milk in Tris Buffered Saline with 0.1% Tween 20 (TBST) and probed with serum, rocking for 16 h at room temperature. After rinsing three times with TBST, the membranes were then incubated with anti-llama horseradish peroxidase (HRP) conjugated secondary antibody (goat anti-llama IgG HRP, ab112786, (Abcam, Cambridge, UK) diluted 1:20,000 in 5% nonfat milk/TBST for 1 h at room temperature. The membranes were washed three times with TBST and visualized with Clarity Western ECL Substrate (Thermo Fisher Scientific Inc., Waltham, MA, USA).

Library Generation. Once a satisfactory result was observed on Western Blot, 200 mL of heparinized blood was collected from the alpaca, and peripheral blood lymphocytes (PBLs) were isolated by gradient centrifugation. An SDAb library was generated using techniques as described [34]. Briefly, total RNA was isolated (RNAeasy; QIAGEN), and cDNA was generated by reverse transcription PCR (Invitrogen). The open reading frames encoding all immunoglobulin heavy chains were amplified by RT-PCR with primers. The SDAb sequences were amplified with nested PCR primers (Forward-1: GTCCTGGCTGCTCTTCTACAAGGC; Reverse-1: GGTACGTGCTGTTGAACTGTTCC; Forward-2: GATGTGCAGCTGCAGGAGTCTGGRGGAGG; Reverse-2: GGACTAGTGCGGCCGCTGGAGACGGTGACCTGGGT) to generate flanking sequences to the framework regions 1 to 4 amenable to restriction endonuclease (R.E.) insertion into pSEX81 surface expression phagemid vector (PR3005, Progen Biotechnik GmbH, Heidelberg, Baden-Wuerttemberg, Germany). PCR products were gel isolated, cut with R.E., vector ligated with T4 DNA ligase, and electro-transformed into competent E. coli TG1 cells. Transformants were grown in 2TY medium containing 2% glucose and 100 μg/mL ampicillin at 37 °C overnight. Transformed bacteria were incubated with hyperphage (PRHYPE, Progen Biotechnik GmbH, Heidelberg, Baden-Wuerttemberg, Germany). Released phage containing SDAb fragments were subjected to multiple rounds of enrichment by bio-panning on PD-1-Fc coated plates.

Antigen Selective Panning of PD-1-specific SDAb Sequences by Phage Display. Phage clones containing canine PD-1 reactive SDAb sequences were selected using phage display and panning protocols as detailed in [35]. Canine PD-1-Fc chimeric protein (SinoBiological. Beijing, China) was coated on 96-well plates, and PD-1 binding phage were enriched by panning for five consecutive rounds. Plate-bound phage were washed, eluted, and used to infect TG-1 cells to generate a sub-library for subsequent rounds of selection. Colonies were scraped, pooled, washed, and pelleted for freezing as glycerol stocks. Briefly, 500 μL of the bacterial sub-library stock was grown in 2x Yeast Extract Tryptone medium (2 × TY) supplemented with 1% glucose, 70 μg/mL kanamycin, and 100 μg/mL ampicillin for 2.5 h at 37 °C, then infected with 1 × 10^12^ helper phage. After incubation, cell debris was removed by centrifugation, and phage particles precipitated by addition of 20% Polyethylene glycol 8000 in 1.5% NaCl *w*/*v*. Precipitated phage were resuspended in PBS and used for rounds of selection on immobilized PD-1-Fc-coated 96-well microtiter plates. Clones expressing anti-PD-1 VHHs were enriched through five rounds of panning.

Screening of PD-1 Selected Phage-Derived SDAb Sequences. Ninety-six clones were selected for affinity testing through Phage ELISA (Enzyme-Linked Immunosorbent Assay—PE ELISA). Each selected clone was cultured in 1 mL Terrific Broth medium (TB) (Invitrogen) containing ampicillin for 3 h and induced with 1 mM IPTG overnight. After osmotic shock, the cell lysates were collected and incubated in microtiter plates coated with canine PD-1-Fc fusion protein (Sino Biological), 100 μg/mL in Phosphate Buffered Saline (PBS) with 1% Fraction V Bovine Serum Albumin (BSA) (Bio-Rad, Hercules, CA, USA). After 1 h, mouse anti-HA antibody as well as goat anti-mouse IgG-alkaline phosphatase were added and incubated successively. Absorbance at 405 nm was determined by microplate reader (Bio-Rad) after addition of alkaline phosphatase. Selected clones were cultured and processed for plasmid isolation (Qiagen) and Sanger sequencing. Subsequent sequence analysis and alignment (SnapGene, San Diego, CA, USA) allowed for grouping of clones into different families according to their amino acid sequence in complementary determining regions (CDRs). At least 30 individual clones were identified and their binding to canine PD-I protein verified and ranked via ELISA binding activity. High activity binders were converted to G-block sequences with optimized codons for bacterial expression and cloned into a pET28 protein expression vector and transformed into BL21 for production and purification of recombinant SDAb protein. High-affinity PD-I binding clones (Kd < 10 nM as calculated via ELISA method [36] were tested to determine the capacity to block binding of PD-1 to its cognate ligand, recombinant canine PD-L1.

Yeast Production and Analysis of SDAb. Anti-PD-1 SDAb sequences were expressed in wild-type *Komagataella phaffii* (Pichia pastoris) (NRRL Y-11430). DNA fragments flush with a custom signal sequence (E0092, Sunflower Therapeutics, Medofrd, MA, USA, PBC) were cloned into a custom vector (pSUN8Z, Sunflower Therapeutics, PBC) that contained a bacterial origin of replication, a methanol-inducible promoter specific to *Komagataella phaffii,* a terminator specific to *Komagataella phaffii*, and a selectable marker. The plasmid was amplified in E. coli and then linearized and transformed into *K. phaffii* cells using electroporation. Stable transformants were obtained via homologous recombination and selected for on appropriate antibiotic-resistant solid media. Strains were cultivated in 24-well deep well plates (3 mL cultivation volume) at room temperature or in 1 L baffled shake flasks (200 mL cultivation volume) at 25 °C. Strains were cultivated in custom chemically defined media. Glycerol-containing medium was used for the first 24 h to build biomass. The medium was then exchanged for methanol-containing medium to promote protein expression. Samples of cultivation supernatant were analyzed for protein expression using SDS-PAGE. SDS-PAGE was carried out under reducing conditions using Novex 12% Tris-Glycine Gels (Thermo Fisher Scientific, Waltham, MA, USA) according to the manufacturer’s recommended protocol and stained using Coomassie Instant Blue Protein Stain (Abcam). SDAb purification was carried out on an AKTA pure purification system (Cytiva, Marlborough, MA, USA) according to the process described previously [37].

Binding and Blocking Analysis of Yeast-Produced STX-1B5 SDAb. To measure the binding of the aglycosylated form of the anti-PD-1 SDAb (STX-1B5), an ELISA with anti-camelid VHH (Genscript, Piscataway, NJ, USA ) antibodies was used. A 1 μg amount of canine PD-1 (cPD-1) (Sino Biologics) was used to coat wells in a 96-well microtiter plate (Immulon plates, Thermo Fisher Scientific, Waltham, MA, USA) overnight. Standard BSA blocking buffer (Thermo Fisher Scientific, Waltham, MA, USA) was used to block coated wells for 2 h at room temperature. The plate was washed with PBS/0.05% Tween 20 (PBST), and the wells were treated with different concentrations (40–0.3 μg/mL) of anti-PD-1 SDAb, along with a non-specific SDAb control, and incubated at 4 °C overnight. After washing, 200 ng of Rabbit anti-camelid VHH (Genscript) was added to each well. As before, biotinylated anti-rabbit (1:20,000) and streptavidin HRP (1:1000) were added to each well, the plate was incubated for 1 h at room temperature, and was washed after addition of each reagent. TMB substrate was added to each well, and OD was read at 450 nM after incubation.

A competitive ELISA was used to determine blocking cPD-L1 binding to cPD-1. Briefly, 1 μg of cPD-1 was coated on a microtiter plate and incubated at 4 °C overnight. After washing with PBS and Standard BSA blocking buffer (Thermo Fisher), 3 μg of recombinant Fc-tagged cPD-L1 mixed with different concentrations (2.5–0.035 μM) of STX-1B5 was added to each of the wells. After washing, an anti-canine PD-1 antibody (Creative BioMart, Shirley, NY, USA (Cat# TAB-028)) was added to the wells, and this was incubated overnight at 4 °C, after which 0.1 μg/mL of anti-Fc IgG antibody was added to each well for an hour at room temperature. The plate was washed, and TMB substrate was added. After incubation, OD was read at 450 nm for SDAb-treated wells and compared with anti-cPD-1 antibody.

Nanobody STX-1B5 and Canine PD-1 Structural Modeling and Complex Interaction Analysis. The STX-1B5:PD-1 complex was predicted using AlphaFold 3, an advanced deep-learning model for protein structure and interaction prediction [38]. The AlphaFold 3 server was used to generate a high-confidence model of nanobody STX-1B5 bound to canine PD-1, incorporating structural constraints to optimize interface complementarity and maintain biologically relevant binding conformations. Protein–protein interactions, including hydrogen bonding, electrostatic contacts, and van der Waals forces, were analyzed using ChimeraX version 1.7.1 [39]. Hydrogen bond formation, hydrophobic interactions, and key contact residues within the C-C′ and F-G loops of PD-1 were identified and mapped. Distance-based analyses were performed to evaluate critical ionic, hydrogen bonding, and van der Waals interactions contributing to STX-1B5 binding affinity. PyMOL 3.1 was used for structural analysis, rendering, and figure preparation [40], including surface mapping of interaction sites and CDR visualization through cartoon representations.

## 3. Results

### 3.1. Construction and Selection of Canine PD-1 SDAb Library

Following the successful elicitation of an antibody response in a healthy alpaca to canine PD-1 utilizing a DNA prime and protein boost immunization strategy, mRNA was extracted from PBLs and utilized to construct a phage expression library. Approximately 30 independent clones were initially selected after five rounds of panning with canine PD-1 protein. Five primary candidates made the final selection after subsequent DNA sequence analysis to remove redundant clones, confirmation of sufficient bacterial protein expression profiles, binding affinity, and blocking activity.

### 3.2. Alternative Host Expression of an Anti-Canine PD-1 SDAb

**Figure 1:**
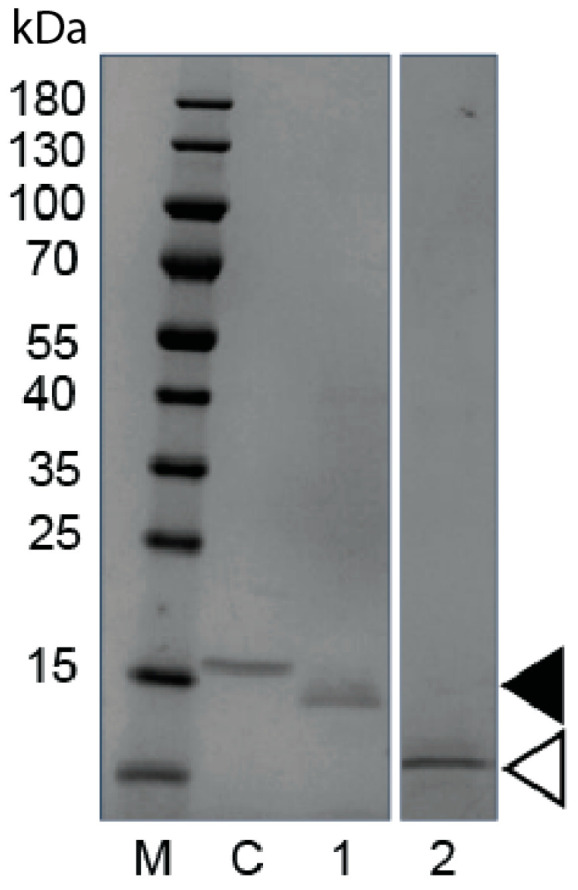
Analysis of Yeast-Expressed Glycosylated and Aglycosylated SDAb STX-1B5. Coomassie-stained SDS-polyacrylamide electrophoresis gel of denatured unpurified cultivation supernatant containing extracellular secreted yeast proteins. M—molecular weight markers in kilodaltons kDa, C—yeast strain expressing a control SDAb, 1—yeast strain expressing original 1B5 (filled arrow), 2—yeast strain expressing aglycosylated STX-1B5 (Open arrow).

The biophysical characteristics of each of the five primary candidates were evaluated to determine which SDAb sequences had the highest feasibility for expression and purification from yeast using a previously developed platform process [37] (Table 1). Candidate 1B5 was selected as the most promising based on its overall isoelectric point (pI) and GRAVY (Grand Average of Hydropathy) scores, and the pI of the SDAb variable region specifically. Initial expression of 1B5 in *K. phaffii* showed secreted protein expression, but expression levels were lower than a control SDAb also expressed in *K. phaffii* (Figure 1, Lanes 1 and C). Smeared banding on the SDS-PAGE indicated that the product may be glycosylated. Expression of a modified protein sequence (STX-1B5) with a single amino acid change at a putative N-linked glycosylation site showed significantly improved expression without any smeared banding (Figure 1, Lane 2). STX-1B5 was purified using the platform process developed in [37], which does not require any purification tags or affinity resins. Host cell DNA and host cell protein were removed to below typically acceptable levels for preclinical development.

### 3.3. Affinity and PDL-1 Blocking Binding of STX-1B5 to PD-1 Validated via ELISA

**Figure 2:**
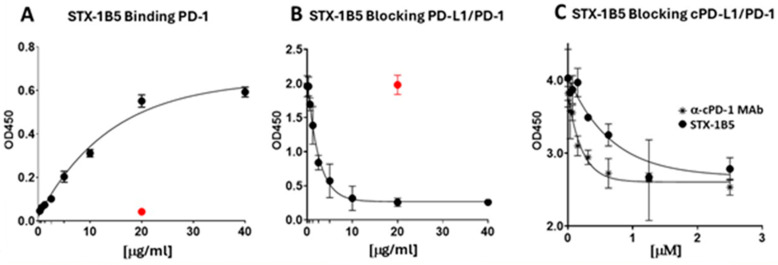
Binding of STX-1B5 to PD-1 and Blockade of PD-1 binding to PD-L1. (**A**). Binding of increasing concentrations of STX-1B5 or a non-specific SDAb (20 μg/mL red dot) to recombinant canine cPD-1 assessed by ELISA using anti-VHH antibodies and HRP-linked anti-IgG antibodies. (**B**). Blocking activity of STX-1B5 (expressed as μg/mL), inhibition of canine PD-L1 binding to canine PD-1 was assessed using competition ELISA, where increasing concentrations of STX-1B5 were combined with 1 μg of cPD-L1 and added to cPD-1 and compared with a previously validated negative control (red dot). (**C**). Blocking activity of STX-1B5 compared to commercially available α-PD-1 Mab (expressed as μM).

STX-1B5 binding to cPD-1 coated on the wells of a microtiter plate was detected using anti-camelid VHH antibodies. STX-1B5 exhibited binding to cPD1 at concentrations as low as 1.2 μg/mL (Figure 2A), as compared with a non-specific SDAb control (20 μg/mL). Maximum binding was seen at 20 μg/mL of STX-1B5. Competition ELISA was used to validate the blocking of cPD-1/cPD-L1 binding by STX-1B5. Canine PD-1 was coated on the wells of a microtiter plate, following which a mixture of recombinant Fc-tagged cPDL-1 and one of various concentrations of STX-1B5 was added to each cPD-1-coated well. Anti-Fc antibodies were used to detect the binding of cPD-L1 to cPD-1. Blocking of cPD-L1 binding was detected in mixtures where the concentration of STX-1B5 was 0.625 μg/mL and greater. Figure 2B demonstrates maximum blocking seen at 10 μg/mL. Similarly, Figure 2C exhibits competition ELISA comparing the blocking of the commercially available α-cPD1 MAb and STX-1B5 expressed in relative concentrations [μM] for each agent. Microtiter plate wells coated with cPD1 were then incubated with a mixture of biotinylated cPD-L1 and one of various equimolar concentrations of α-canine MAb or STX-1B5. Binding of PD-L1 was detected using streptavidin-HRP, followed by the addition of an ECL substrate.

### 3.4. Nanobody Binding Interface and Overlap with PD-L1/PD-L2 Sites

**Figure 3:**
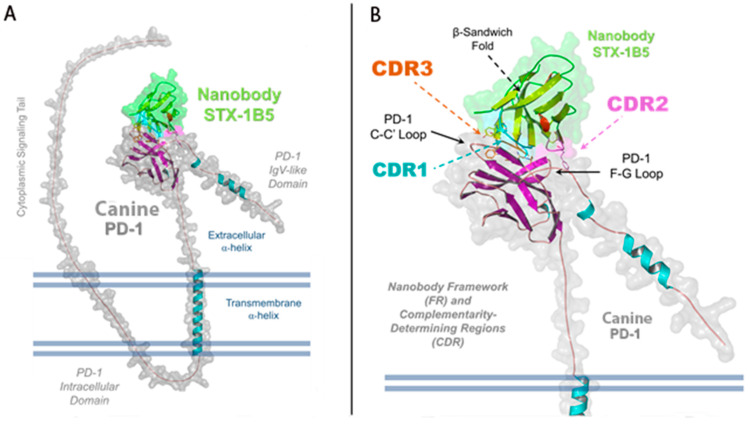
Structural Representation of STX-1B5 Bound to Canine PD-1. Illustration of a refined AlphaFold 3 model of the nanobody STX-1B5 (green) interacting with canine PD-1 (gray). (**A**) The full-length structure of PD-1 is shown, highlighting key domains, including the IgV-like extracellular domain and extracellular α-helix, the transmembrane α-helix, and the N-terminal, intracellular signaling tail. The nanobody binds within the shallow groove of PD-1’s extracellular domain, engaging both the C-C′ and F-G loops and the central β-sheet platform, the primary site for PD-L1/PD-L2 recognition. (**B**) A zoomed-in view of the binding interface highlights the three complementarity-determining regions (CDRs) of the nanobody: CDR1 (cyan), CDR2 (magenta), and CDR3 (orange). The nanobody framework (FR) is also labeled, demonstrating the structural stability provided by the immunoglobulin β-sandwich fold. The surface representation of STX-1B5 emphasizes its interaction footprint on PD-1, while dashed lines indicate the regions of contact mediated by each CDR loop.

**Figure 4:**
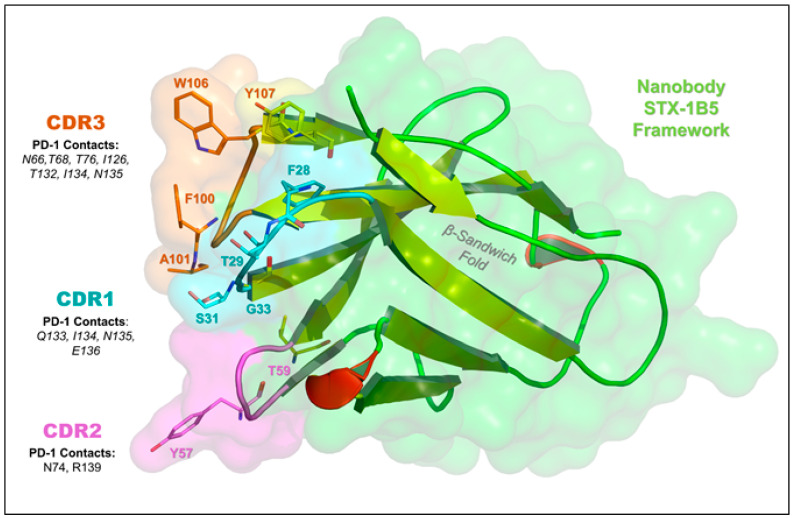
Key Amino Acid Interactions in the STX-1B5:PD-1 Binding Interface. This figure highlights the nanobody STX-1B5 (green) and its complementarity-determining regions (CDRs) responsible for PD-1 binding in greater detail. CDR1 (cyan), CDR2 (purple), and CDR3 (orange) interact with the central β-sheet platform and C-C′ and F-G loops of PD-1, which is a key interface for PD-L1 and PD-L2 binding. The β-sandwich fold of the nanobody is shown (yellow cartoon arrows), providing structural support for antigen recognition by the CDRs. We identified several key nanobody residues that mediate interactions with canine PD-1, including F28, T29, S31, G33 (CDR1); T59, Y57 (CDR2); and F100, A101, W106, Y107 (CDR3). Important PD-1 residues involved in nanobody binding—F56, T59, D61, E136, and Y137—overlap with the PD-L1/PD-L2 binding site and are also targeted by pembrolizumab. This suggests that STX-1B5 may competitively inhibit PD-1 signaling, mimicking the effects of checkpoint inhibitors.

**Figure 5:**
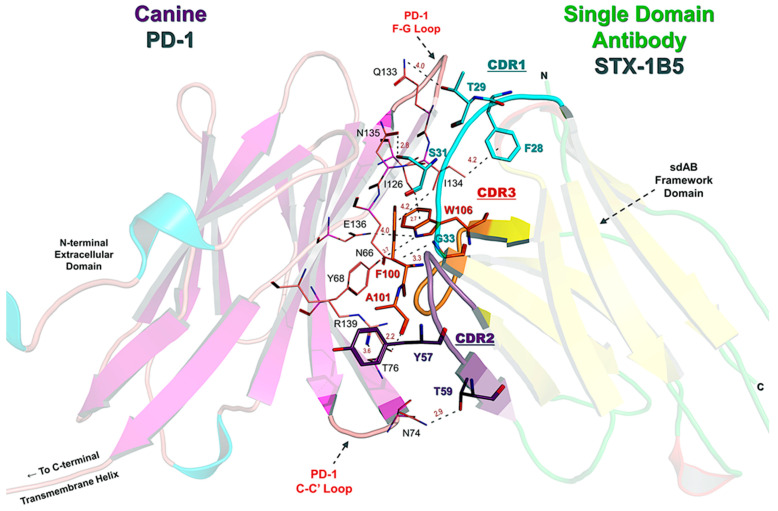
Detailed Amino Acid Contacts in the STX-1B5:PD-1 Binding Interface. This figure provides a detailed structural view of the STX-1B5 nanobody (right, transparent cartoon) bound to canine PD-1 (left, transparent cartoon), highlighting key contact residues. The CDRs of STX-1B5 are distinctly colored: CDR1 (cyan), CDR2 (purple), and CDR3 (orange), with residues within each CDR colored accordingly. Key nanobody residues (bold black text) directly interacting with PD-1 include: F28, T29, S31, G33 (CDR1); Y57, T59 (CDR2); and F100, A101, W106, Y107 (CDR3). These residues form hydrogen bonds and hydrophobic contacts with PD-1 in the binding cleft formed between the C-C′ and F-G loops, the primary binding sites for PD-L1 and PD-L2. PD-1 residues involved in direct nanobody interaction (maroon text) include N66, Y68, T76, N74, I126, Q133, N135, E136, and R139, reinforcing key hydrophobic and electrostatic interactions at the binding interface. Hydrogen bond distances and van der Waals contacts are labeled in gray, emphasizing the close-range interactions that stabilize the complex.

STX-1B5 binding to cPD-1 was modeled using AlphaFold 3, which enables high-confidence structural predictions without requiring crystallographic or cryo-EM data [39]. The binding interface occurs at the C-C′ and F-G loops of cPD-1, a region that partially overlaps with the binding site for PD-L1 and PD-L2 (Figure 3). STX-1B5 is modeled to bind cPD-1 via three complementarity-determining regions (CDRs; see Figure 4), which interact directly with specific cPD-1 residues through a combination of hydrogen bonding, hydrophobic contacts, and backbone interactions, as detailed in Figure 5. For dog cPD-1, our model predicts that CDR1 is comprised of amino acid residues F28, T29, S31, and G33 (highlighted in black text in Figure 5), which make direct interactions with cPD-1 residues I126 and N135. These contacts help stabilize the β-sheet core adjacent to the F-G loop, reinforcing nanobody binding. CDR2 (purple) includes residues Y57 and T59, which interact primarily with cPD-1 residues R139 and N74. These contacts bridge β-sheet elements with flexible loop regions, forming additional points of stabilization. CDR3 (orange) is predicted to extend deeply into the cPD-1 interface, with W106, F100, and A101 engaging PD-1 residues Y68, N66 and E136 through a mix of hydrophobic and polar interactions. These contacts overlap significantly with cPD-L1/L2 binding sites, reinforcing the potential competitive inhibition by STX-1B5.

### 3.5. Structural Comparisons with Pembrolizumab (Keytruda)

The development of checkpoint inhibitors targeting PD-1 has led to the widespread clinical use of monoclonal antibodies (MAbs) such as pembrolizumab, which effectively block PD-1 interactions with PD-L1 and PD-L2, restoring T-cell activation in cancer immunotherapy. Given that STX-1B5 binds overlapping regions of cPD-1, a comparison with pembrolizumab helps contextualize its binding mode and therapeutic potential despite fundamental differences in molecular architecture and size.

Both STX-1B5 and pembrolizumab engage the C-C′ and F-G loops of cPD-1, key regions for cPD-L1 binding [13], though their specific interactions differ. However, pembrolizumab, as a full-length IgG (~150 kDa), engages a broader surface area on PD-1, whereas STX-1B5 (~15 kDa), as a single-domain nanobody, achieves specificity through a more compact binding interface. While both inhibitors interact with residues in the central β-sheet platform of PD-1, their binding modes and interaction networks differ.

Notably, STX-1B5 forms a hydrophobic triad involving CDR1 (F28) and CDR3 (Y107 and I134) that reinforces binding stability at the cPD-1 interface. In addition, STX-1B5 engages key cPD-1 residues, including Q133, I134, and E136, which are also contacted by pembrolizumab but may be stabilized differently due to the distinct molecular framework of the nanobody (Figure 5). Furthermore, ionic interactions between E136 and R139, along with hydrogen bonding involving T132 and N135, contribute to STX-1B5’s strong binding affinity within the ligand-binding surface.

## 4. Discussion

To the best of our knowledge, this study is the first to report on the generation and functional characterization of a yeast-produced, Alpaca-derived single-domain antibody (SDAb) targeting the cPD-1 protein. This antibody, STX-1B5, provides an immediate candidate to address the need for economical, species-specific cancer immunotherapy for use in dogs. It functions similarly to existing human MAb therapies, interacting with the canine PD-1 molecule at overlapping, but distinct, points as those medications, and effectively blocking PD-1/PDL-1 interaction. As such, it shows promise either as monotherapy for checkpoint inhibition or potentially in combination with other checkpoint inhibitors.

This study employed non-mammalian cell culture platform technologies to develop therapeutic nanobodies against canine PD-1, aiming to create novel, low-cost immunotherapies for treating canine cancer. To isolate anti-PD-1 SDAb, we immunized an Alpaca rather than generate a naïve library, as immunization results in higher affinity and specificity to antigens because of clonal selection and affinity maturation. The choice of phage display and biopanning as methods of library selection was based on the speed and relative ease of this method, as well as the iterative nature, resulting in high-affinity binders. This approach yielded several candidates (Table 1), but STX-1B5 clearly outperformed the others in its adaptability to yeast-based production. This was an important step to meet our goal to develop an affordable therapeutic for the veterinary market: yeast-based production can enable significant cost advantages compared to mammalian or bacterial hosts due to fast growth, secretion of properly folded proteins, no contamination with endotoxin or viruses, and lower impurity levels [41,42]. The SDAb manufacturing process deployed here is also compatible with end-to-end continuous production platforms, leading to additional potential savings in the cost of goods manufactured [43].

This study highlights the gap between the identification of an SDAb candidate by simple affinity screening and the optimized production of a potential therapeutic agent. We identified 30 initial candidates that were narrowed to 5 after initial testing, but ultimately, 1 finalist. Sequence redundancy, inferior activity, and poor producibility all led to the dismissal of certain candidates. Even our most successful candidate required modification to remove a potential N-linked glycosylation site, after which STX-1B5 was successfully expressed in *K. phaffii* (P. pastoris) and purified using a simple, two-column straight-through chromatography process without the need for protein tags or affinity resins. Whereas the traditional manufacturing approach and scale-up for proteins utilizes mammalian cell expression systems, the use of alternative organisms, such as yeast, has been determined to enable greater speed and ease of use compared to standard mammalian cell production [30], plus lower cost processes [44] and accelerated development timelines [45].

The structural comparison between STX-1B5 and pembrolizumab (Keytruda) highlights key advantages of the SDAb platform over MAb. STX-1B5 has a substantially smaller molecular footprint (~15 kDa) than pembrolizumab (~150 kDa), a full-length IgG monoclonal antibody. The SDAb should exhibit stable PD-1 binding with smaller steric hindrance. Its compact structure may also enhance tissue penetration, biodistribution, and accessibility to dense tumor microenvironments, and its lack of an Fc region should decrease the likelihood of an adverse immunologic event, regardless of the species of use. The smaller molecule lends itself to microbial production, with much lower costs than the use of mammalian cell lines frequently used to produce MAb. Beyond its immediate application in PD-1 blockade, STX-1B5 represents a broader opportunity for SDAb-based immunotherapy. Its small size, high stability, and tunable binding properties make it an ideal candidate for bispecific or multivalent therapeutic formats, potentially improving immune modulation strategies in cancer and other diseases. Future studies will be necessary to validate these structural insights through functional assays and assess STX-1B5’s potential as a next-generation immunotherapeutic agent. These distinctions suggest that STX-1B5 functions as an effective PD-1 inhibitor, offering potential advantages over monoclonal antibodies due to its smaller size, unique binding scaffold, and potential for engineering into multivalent or bi-specific therapeutics [46]. Given the versatility of nanobody technology, STX-1B5 could serve as a therapeutic platform for immune checkpoint blockade strategies beyond traditional PD-1-targeting Mabs.

## 5. Conclusions

The first and necessary step to developing any Immune Checkpoint Inhibitor is effective disruption of the targeted receptor/ligand complex. We sought to produce the first anti-canine PD-1 SDAb with functional activity to block canine PD-1/PDL-1 binding. By molecular modeling, the STX-1B5 sequence was shown to interact with the region of canine PD-1 required for binding to the cognate PDL-1 receptor. Empirical evidence for potential therapeutic capacity was demonstrated in blocking and binding studies with canine PD-1 and canine PD-L1 recombinant proteins.

The recombinant SDAb protein, STX-1B5, was produced in a yeast expression system, as success in the clinic requires not only therapeutic functionality but also affordability. Production and regulatory costs are among the two critical factors that impact the development of biologic-based therapeutics. Production of a biologic agent in a yeast expression system is considerably less expressive than traditional mammalian cell culture methods. Not only are material and personnel costs dramatically reduced, but utilization of yeast culture avoids much of the regulatory cost burden involved in testing, qualifying, and maintaining pathogen-free mammalian cell cultures and media constituents commonly used in ICI production.

The cost of therapy is also impacted by the level of care required for the administration of biologics, which are generally infused via the intravenous route. Trained individuals and specialized equipment for sedation, infusion, observation, and recovery stages are required over a several-hour period in a clinical setting. In contrast, SDAb can be administered via subcutaneous injections, where, due to their small size, SDAb will diffuse into the bloodstream, circumventing the need for complex clinical care.

## 6. Patents

A portion of the work described here was used for the filing, which resulted in the United States Patent Application Publication Mourich et al. (10) Pub. No.: US 2021/0047412 Al (43) Pub. Date: 18 February 2021.

## Figures and Tables

**Table 1:** Protein Sequence Analysis of Primary Candidate SDAbs. Calculated molecular weight in kilodaltons (kDa), isoelectric point (pI) for the total protein sequence and for the variable regions (CDR1–3), and grand average of hydropathy (GRAVY) values for each SDAb protein sequence. The GRAVY value is defined by the sum of hydropathy values of all amino acids divided by the protein length.

Candidate SDAb	MW (kDa)	Overall pI	Variable Region pI	Overall GRAVY
1B5	12.3	7.94	3.67	−0.42
5A5	12.2	9.04	9.96	−0.31
4B2	12.4	6.80	3.80	−0.27
5A1	12.7	4.44	3.17	−0.40
4B4	12.8	5.78	4.50	−0.55

## Data Availability

The original contributions presented in this study are included in the article. Further inquiries can be directed to the corresponding author(s).
